# Comparison of Twin Screw Derotation Type Versus Single Helical Blade Type Cephalomedullary Nail in the Management of Unstable Intertrochanteric Fractures

**DOI:** 10.7759/cureus.61638

**Published:** 2024-06-04

**Authors:** Varun Thusoo, Brahmpreet Singh Nagpal, Sachin Kudyar, Arjun S Chakrapani, Eshaan Singh Saini, KV Alok, Rahul Pathanaboina, Najeeb Palakkal

**Affiliations:** 1 Department of Orthopaedics, Adesh Medical College and Hospital, Ambala Cantt, IND; 2 Department of Orthopaedics, Government Medical Hospital, Jammu, IND; 3 Department of Orthopaedics, Apollo Speciality Hospitals, Perungudi, Chennai, IND; 4 Department of Orthopaedics, Osmania Medical College, Hyderabad, IND; 5 Department of Orthopaedics, Government Medical College, Jagtial, Hyderabad, IND

**Keywords:** intertrochanteric fracture, cephalomedullary nail, derotation, pfna2, implants, tad

## Abstract

Background: The intertrochanteric fracture is a frequently occurring fracture, often attributed to osteoporosis in older populations. Recently, there has been a proposal to perform early surgical fixation on elderly patients to facilitate early rehabilitation. This approach has been shown to have a beneficial effect in lowering comorbidities. The study aims to compare the efficacy of the twin screw derotation type cephalomedullary nail with that of the single helical blade type cephalomedullary nail in the management of unstable intertrochanteric fractures.

Methodology: The research sample included patients from the orthopedic outpatient and emergency departments of Adesh Medical College and Hospital, Ambala Cantt, India, who were scheduled for surgery for unstable intertrochanteric femur fractures. The patients were categorized into two groups according to the kind of implant they were given: either a twin screw derotation cephalomedullary nail or a single helical blade cephalomedullary nail. The functional result was evaluated by comparing the modified Harris hip score (HHS). Patients with unstable intertrochanteric fractures, including reverse oblique fractures and fractures with posteromedial comminution, as well as patients who provided consent, were included in this study.

Results: Thirteen individuals received treatment with proximal femoral nail antirotation (PFNA2), whereas 19 individuals received treatment with proximal femoral nail (PFN). The mean age in the PFNA2 group was 69.51, whereas the mean age in the PFN group was 70.804. There were three patients in the PFNA2 group and five patients in the PFN group who had a tip apex distance of more than 25 mm. According to the Cleveland index, nine patients in the PFNA2 group and eight patients in the PFN group had an implant location that was not optimum. Four patients in the PFNA2 group and seven patients in the PFN group had a neck shaft angle difference of more than 10° between their undamaged and operated sides. The mean HHS was 74.55 for the PFNA2 group and 69.88 for the PFN group. The PFNA2 group exhibited four problems, whereas the PFN group had five issues.

Conclusion: The study found that both implants offer similar functional outcomes, with adherence to specific radiological parameters optimizing results. While both face similar challenges with osteoporosis, there was no notable distinction between them. Notably, the PFNA2 group showed superior outcomes in perioperative morbidity.

## Introduction

The intertrochanteric fracture is a frequently occurring fracture, often attributed to osteoporosis in older populations. Recently, there has been a proposal to perform early surgical fixation on elderly patients to facilitate early rehabilitation and effectively reduce comorbidities [1-4]. Femoral pertrochanteric fractures have a significant death rate during the first year after their occurrence. In cases of unstable fractures, intramedullary implants often provide biomechanical benefits compared to extramedullary implants. The dynamic hip screw, which is widely regarded as the most effective therapy for stable fractures, has been deemed unsuitable for treating the unstable category of intertrochanteric fractures [5,6].

The optimal technique for stabilizing unstable fractures involves using an intramedullary nail in conjunction with a dynamic implant for femoral head/neck stability [7]. Over time, other nail designs that include either a single compression screw or a combination of a compression screw and an antirotating screw, such as the proximal femoral nail (PFN), have gained popularity for the treatment of unstable fractures. Biomechanical research has shown that the helical blade, when implanted without reaming, has enhanced resistance to rotation and varus collapse due to the compaction of the cancellous bone surrounding it [8].

The entrance location is crucial for achieving appropriate reduction, reliable fixation, and preventing problems linked to the implant [9]. Research has proposed that the lateral entrance site during the reaming of intramedullary nail insertion might harm the gluteus muscular tendon. According to research on the architecture of the greater trochanter, the best location for the entry point is at the posterior tip. This is to ensure that the implant can fit well in the curved section of the proximal femoral medullary canal [10].

To provide a high standard of fixation and minimize harm to the gluteus medius, it is recommended that the entrance site for proximal femoral nail antirotation (PFNA2) be positioned 5 mm toward the center from the tip of the greater trochanter. The incidence of lateral cortical impingement was higher in instances with lateral entrance points than in those with medial entry. According to clinical research, intertrochanteric fractures have been shown to have worse results in individuals with osteoporosis. Consequently, several techniques, such as cement reinforcement and advancements in implant structure, are being used to enhance stability in osteoporotic intertrochanteric fractures. The ongoing discussion is on the selection of helical blade and lag screw fixation [11].

Several studies indicate that there is no discernible difference in clinical effectiveness, except for the extended duration of the surgical procedure linked to the use of lag screws. Nevertheless, biomechanical research has shown that helical blades enhance stability [12,13]. Helical blades have been associated with complications such as "cut-out" and "cut-through" rates. The most advantageous selection between the two screws remains ambiguous, necessitating more exploration [14,15]. Therefore, the study aims to compare the efficacy of the twin screw derotation type cephalomedullary nail with that of the single helical blade type cephalomedullary nail in the management of unstable intertrochanteric fractures.

## Materials and methods

Study design and participants

The research was conducted at the orthopedics department at Adesh Medical College and Hospital, Shahbad, Ambala Cantt, India. It was a prospective, randomized, controlled trial from July 2023 to April 2024. The research sample included patients from the orthopedic outpatient and emergency departments of Adesh Medical College and Hospital who were scheduled to have surgery for unstable intertrochanteric femur fractures.

Selection criteria

The study's inclusion criteria involve individuals over 30 years old with unstable intertrochanteric fractures, including reverse oblique fractures and fractures with posteromedial comminution. Additionally, patients must have provided consent to participate. Conversely, the exclusion criteria encompass individuals below 30 years of age, those with stable fractures, and fractures older than one month.

Data sources and variables

The patients were categorized into two groups according to the kind of implant they were given: either a twin screw derotation cephalomedullary nail or a single helical blade cephalomedullary nail. The functional result was evaluated by comparing the modified Harris hip score (HHS). The surgical operation was conducted on a fracture table while the patient was under spinal anesthesia. Both groups received preoperative antibiotics. The duration of the surgery, the quantity of image intensifier shots used, and the amount of blood lost were documented. Isometric quadriceps and ankle pumps were started promptly.

Patients were ambulated starting on the second day after surgery, and partial weight bearing was permitted using a walker. Additional weight bearing was permitted depending on the development of bone healing. Both groups followed a comparable rehabilitation plan; no thromboprophylaxis was administered after the surgery. Evaluations and subsequent monitoring were performed at the six-month and one-year intervals. Clinical and radiological evaluations were conducted at the one-year follow-up, and the HHS was used for functional assessment. Both groups received medical treatment for osteoporosis.

Statistical analysis

The master graphic containing the data was filled out using Microsoft Excel (Microsoft Corporation, Redmond, WA). SPSS Version 20 (IBM Corporation, Chicago, IL, USA) was used to evaluate the data gathered. Frequency, percentage, and mean analyses were employed to characterize the data in descriptive statistics for categorical and continuous variables. The data analysis was conducted using the Mann-Whitney U test for continuous variables and the chi-square test for categorical variables. A statistically significant p value was defined as one that was less than 0.05.

## Results

Table 1 provides an overview of the gender and age distribution among the participants. Regarding gender, most participants were male, constituting 62.5% of the total, while females accounted for 37.5%. Regarding age, the participants were primarily distributed across various age groups. The highest proportion fell within the 50-60 age bracket, comprising 46.86% of the total, followed by those aged 40-50, representing 18.75%. Additionally, individuals aged 60-70 and above 70 each constituted 15.64% and 18.75%, respectively. The mean age of the participants was reported as 60 years, with a standard deviation of ±30 years, indicating a wide range of age variability within the study population.

**Table 1:** Gender and age of the participants

Parameter		Number	Percentage (%)
Gender	Male	20	62.5%
	Female	12	37.5%
Age	40-50	6	18.75%
	50-60	15	46.86%
	60-70	5	15.64%
	Above 70	6	18.75%
	Mean	60 ± 30	

**Figure 1:**
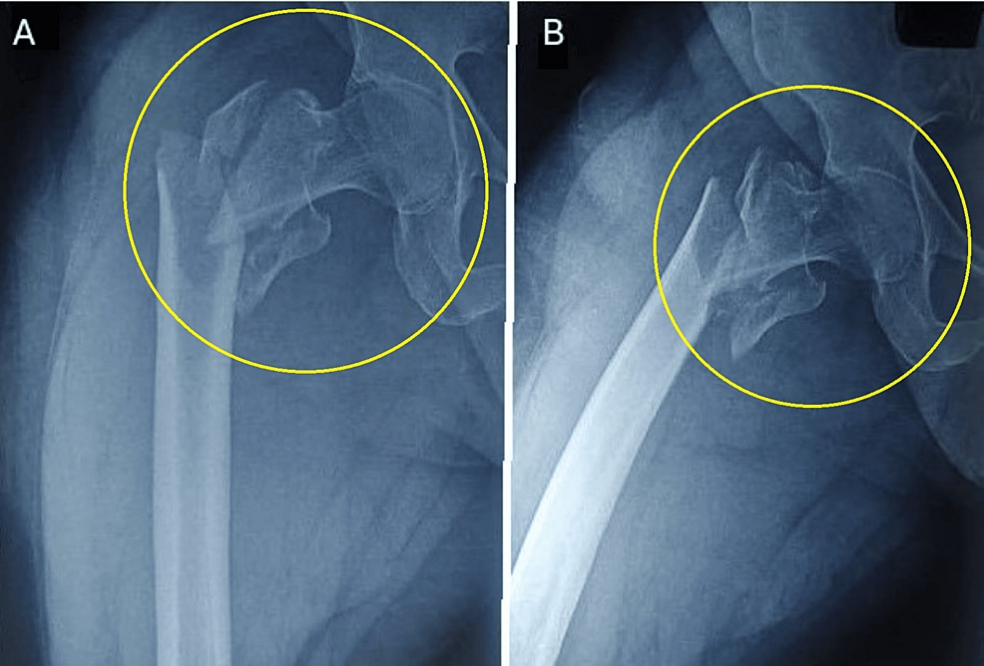
Patient with intertrochanteric fracture. (A,B) X-ray images of a patient with intertrochanteric fracture from two different angles (circles)

Table 2 presents the length of hospital stay and the participants' mean surgery duration. The time between admission and surgery was recorded as two days, while the average length of hospital stay was approximately 4.95 days. Additionally, the mean duration of surgery was reported as 60 minutes. Figure 1 depicts a patient with an intertrochanteric fracture.

**Table 2:** Length of hospital stay and mean duration of surgery

Parameter	Duration
Time between admission and surgery	2 days
Length of hospital stay	4.95 days
Mean duration of surgery	60 minutes

Table 3 displays the tip apex distance (TAD) categorized by different parameters, including PFNA2 and PFN. For PFNA2, two participants (15.38%) had a TAD of less than 20 mm, eight participants (61.54%) fell within the 20-25 mm range, and three participants (23%) had a TAD greater than 25 mm. Similarly, for PFN, two participants (10.53%) had a TAD of less than 20 mm, 12 participants (63.16%) fell within the 20-25 mm range, and five participants (26.31%) had a TAD greater than 25 mm.

**Table 3:** Tip apex distance PFNA2: proximal femoral nail antirotation; PFN: proximal femoral nail

Parameter	PFNA2 = 13		PFN = 19		
	Number	Percentage (%)	Number		Percentage (%)
<20 mm	2	15.38%	2	10.53%	
20-25 mm	8	61.54%	12	63.16%	
>25 mm	3	23%	5	26.31%	

Table 4 presents the Cleveland index categorized by PFNA2 and PFN parameters. Among PFNA2 participants, four individuals (30.77%) were in the optimal position, while nine participants (69.23%) were in a suboptimal position. In contrast, among PFN participants, 11 individuals (57.89%) were in the optimal position, and eight participants (42.11%) were in a suboptimal position.

**Table 4:** Cleveland index PFNA2: proximal femoral nail antirotation; PFN: proximal femoral nail

Parameter	PFNA2 = 13		PFN = 19	
	Number	Percentage (%)	Number	Percentage (%)
Optimal position	4	30.77%	11	57.89%
Suboptimal position	9	69.23%	8	42.11%

Table 5 illustrates the neck shaft angle categorized by PFNA2 and PFN parameters. Among PFNA2 participants, two individuals (15.38%) had a neck shaft angle of less than 5 mm, seven participants (53.85%) fell within the 5-10 mm range, and four participants (30.77%) had a neck shaft angle greater than 10 mm. Similarly, among PFN participants, five individuals (26.32%) had a neck shaft angle of less than 5 mm, seven participants (36.84%) fell within the 5-10 mm range, and seven participants (36.84%) had a neck shaft angle greater than 10 mm.

**Table 5:** Neck shaft angle PFNA2: proximal femoral nail antirotation; PFN: proximal femoral nail

Parameter	PFNA2 = 13		PFN = 19	
	Number	Percentage (%)	Number	Percentage (%)
<5 mm	2	15.38%	5	26.32%
5-10 mm	7	53.85%	7	36.84%
>10 mm	4	30.77%	7	36.84%

**Figure 2:**
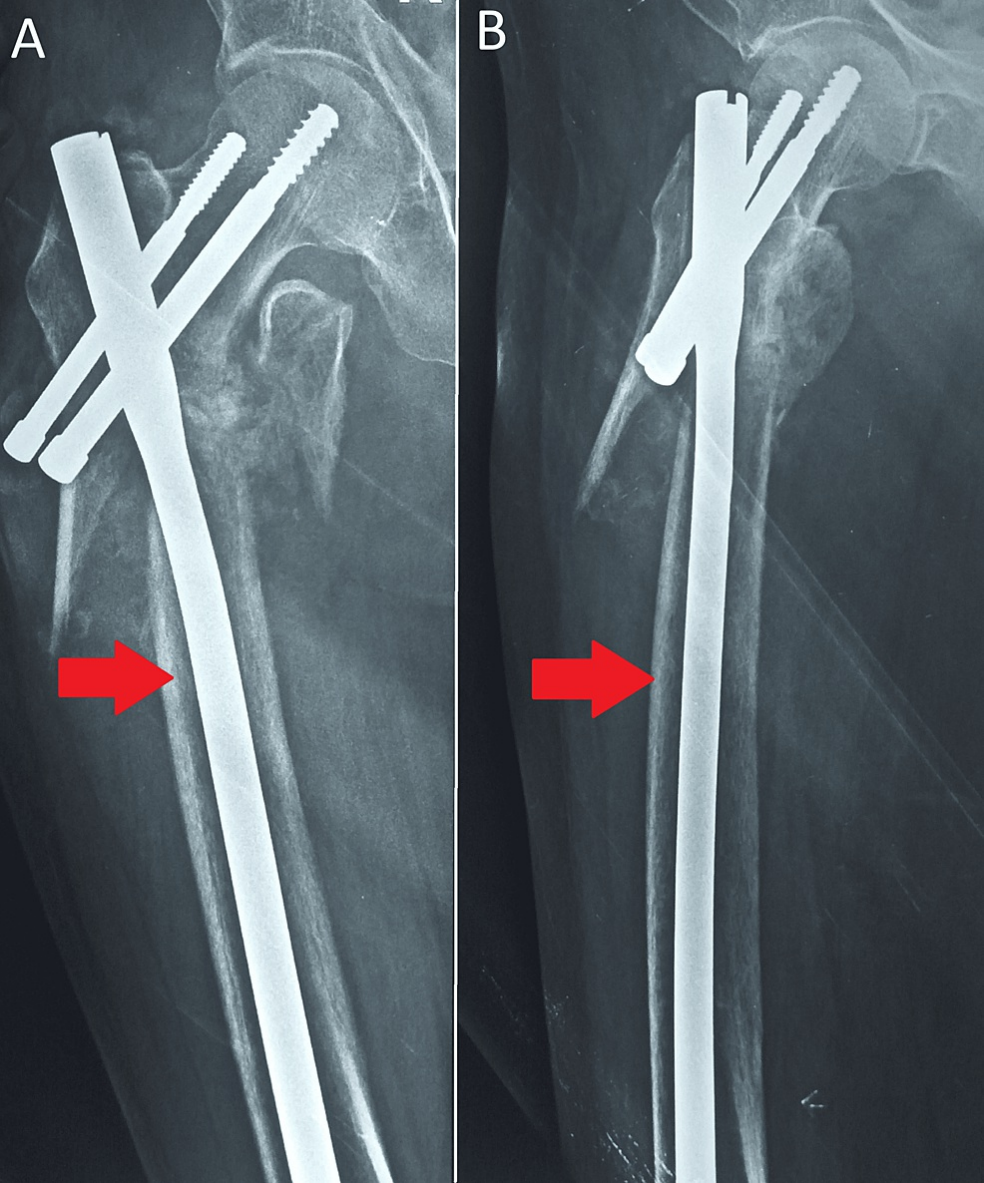
(A,B) Patient with PFN implants (red arrows) PFN: proximal femoral nail

The mean HHS at different postoperative time intervals was calculated, comparing PFNA2 and PFN outcomes. At the one-year mark, the HHS for PFNA2 was 74.55, while for PFN, it was 69.88. The corresponding p value was 0.102. Figure 2 shows a patient with PFN implants.

Table 6 presents the occurrence of complications among participants with PFNA2 and PFN implants, along with corresponding p values. Both PFNA2 and PFN groups experienced one case each of broken implants and Z-effect complications, with no significant difference observed (p = 0.44). Additionally, the occurrence of screw cut-out, revision surgery, and wound infection complications was reported as 0 and 1 for PFNA2 and PFN groups, respectively. The total number of complications was four for PFNA2 and five for PFN, without a statistically significant difference between the groups.

**Table 6:** Complications PFNA2: proximal femoral nail antirotation; PFN: proximal femoral nail P value <0.05 is considered to be significant

Parameter	PFNA2 = 13	PFN = 19	P value
Broken implant	1	1	0.44
Z-effect	1	1	
Screw cut-out	0	1	
Revision surgery	1	1	
Wound infection	1	1	
Total	4	5	

## Discussion

The study included a total of 32 participants, with 20 men and 12 women, aged between 30 and 95 years, and with Singh's index less than 3. The average age of the patients was 60 ± 3 years. Uncomplicated falls were the predominant cause of injury. The mean duration from admission to surgery was two days, whereas the mean duration of hospitalization was 4.95 days. The average time of operation was 60 minutes, and the death rate within one year was 20%. Thirteen individuals received treatment with PFNA2, whereas 19 individuals received treatment with PFN. The mean age in the PFNA2 group was 69.51, whereas the mean age in the PFN group was 70.804.

There were three individuals in the PFNA2 group and five patients in the PFN group who had a TAD of more than 25 mm. According to the Cleveland index, nine patients in the PFNA2 group and eight patients in the PFN group had an implant location that was not optimum. Four patients in the PFNA2 group and seven patients in the PFN group had a neck shaft angle difference of more than 10° between their undamaged and operated sides. The mean HHS was 74.55 for the PFNA2 group and 69.88 for the PFN group. The PFNA2 group exhibited four problems, whereas the PFN group saw five issues.

Werner-Tutschku et al. were the first to describe the occurrence of the Z-effect. They documented a series of 70 fracture cases that were treated with PFN. The Z-effect refers to the horizontal movement of the lower screw, inward collapse of the fracture, and penetration of the femoral head by the upper screw. The reverse Z-effect refers to the horizontal movement of the upper screw toward the side while the lower screw moves toward the center [16]. These authors also recommended that while fixing the fracture, a cervicodiaphyseal angle of less than 125° increases the likelihood of the Z-effect and reverse Z-effect, as well as the risk of the screw causing the femoral head to detach. The reason for this problem has been elucidated as the varus collapse of the fracture and the absence of medial cortical support [17].

When the bone density of the femoral neck was lower than that of the femoral head, as is often seen in unstable fractures, there was a propensity for the inferior screw to migrate. Strauss et al. also advised against using femoral nails with two interlocking head screws in situations of fractures with severe comminution and medial support loss [17]. Devices equipped with helical blades are specifically developed to enhance rotational stability, conserve the bone material of the femoral head, and avoid varus collapse. These devices are inserted with impaction toward the femoral head. While these implants provide enhanced biomechanical stability compared to traditional PFN, they are nonetheless susceptible to complications [18]. Therefore, Brunner et al. have reported three instances of helical blade devices causing perforation in the femoral head. These cases occurred in patients who had achieved successful fracture reduction and appropriate location of the implant [19]. These authors recommend avoiding the placement of the blade less than 5 mm below the joint in cases of severe osteoporosis to prevent perforation of the femoral head.

Implementing the PFNA2 included replacing the previous blade with a helical one. This alteration aimed to decrease the probability of cut-out and completely prevent the occurrence of the Z-effect failure mode that was present in the prior PFN. Multiple biomechanical investigations have provided evidence supporting this claim [20]. However, the occurrence of cut-out has not been eradicated and remains the most prevalent kind of failure. In biomechanical research conducted by Born et al., a comparison was made between threaded screw and helical blade constructions in a model of pertrochanteric fracture repair utilizing polyurethane femoral heads. The study revealed that the blade device is more susceptible to cut-out, as shown by the results [20]. Pu et al. found that the average TAD was 16.8 mm in their study. However, they recommended maintaining a minimum distance of 10 mm between the helical blade tip and the subchondral bone to prevent skull perforation. They also proposed employing a shorter blade for this purpose [21].

Nikoloski et al. argue against using the TAD rule of less than 25 mm for the PFNA2 [22]. It is recommended to avoid using a TAD with a length of less than 20 mm to prevent the risk of axial cut-out (medial migration). Similarly, avoiding using a TAD longer than 30 mm is advised to prevent the risk of cephalad cut-out. The helical blade was innovated due to its biomechanical advantages in the context of osteoporosis [13]. The blade may be inserted without the need for reaming, therefore maintaining the essential bone stock in the femoral head. During the process of insertion, it compresses the cancellous bone around it, resulting in improved stability and resistance to both varus collapse and rotational stress [23]. The PFNA2's flexible tip mitigates bone stress, resulting in reduced implant failure, namely distal nail breakage and distal locking screw breakage. The findings indicated that both implants were efficacious; however, PFNA2 exhibited many benefits including less radiation exposure, shorter duration of surgery, decreased blood loss, and fewer complications [24]. As a result, the healing and rehabilitation process was expedited. PFNA2 is increasingly used as the primary device for treating unstable intertrochanteric fractures in older patients and may be used for younger patients [25,26].

Limitations of the study

The study has several limitations. First, the sample size was relatively small, comprising only 32 individuals, which could restrict the generalizability of the findings. Second, the study focused on individuals aged between 30 and 95 with a Singh's index of less than 3, potentially excluding younger patients and those with higher Singh's index values. Additionally, the study primarily included patients with uncomplicated falls as the predominant cause of injury, limiting the diversity of the patient population. Moreover, while the average time from admission to surgery and the mean duration of hospitalization were reported, specific factors influencing these durations were not explored.

## Conclusions

The study concluded that both implants offer comparable functional outcomes. Adhering to specific radiological parameters, such as maintaining a TAD below 25 mm and achieving a center-center position on the Cleveland index, may optimize functional results. Despite encountering similar challenges in addressing osteoporosis, no significant difference was observed between the two implants. Notably, the PFNA2 group exhibited superior outcomes concerning perioperative morbidity.
